# Cultures of Harm in Institutions of Care: Introduction

**DOI:** 10.1093/shm/hky103

**Published:** 2018-11-19

**Authors:** Louise Hide, Joanna Bourke

**Affiliations:** Department of History, Classics, and Archaeology, Birkbeck, University of London, Malet Street, London, UK

In February 2015, the ‘Lampard Report’ was published following investigations that were carried out in Britain by the Department of Health and three NHS hospitals into the late ‘famous, flamboyantly eccentric, narcissitic [*sic*] and manipulative television personality’ Jimmy Savile.^1^ During the same year, the Independent Inquiry into Child Sexual Abuse was established to consider the growing evidence of institutional failures to protect children from child sexual abuse in England and Wales. Two years earlier, the ‘Francis Report’, which was based on oral hearings and over 1 million pages of ‘raw material’, detailed the serious failings of care at the Mid Staffordshire NHS Foundation Trust.^2^ Just a few months before that the Department of Health published its response to the exposure of cruel and abusive practices at Winterbourne View Hospital which provided long-term residential care for people with intellectual disabilities.^3^ Simultaneously, other major inquiries were taking place across the UK as well as in Australia and other parts of the world.^4^

These were the latest inquiries in a raft of investigations into abuse in hospitals and care facilities that had been taking place in England for over 40 years. The first was triggered by press exposés and the tireless campaigning of Barbara Robb, who established the pressure group Aid for the Elderly in Government Institutions (AEGIS) in the mid 1960s. Her book, *Sans Everything. A Case to Answer* (1967) was an important step towards revealing the dehumanising conditions in which people were being kept in long-term psychiatric ‘back’ wards and hospitals for people with ‘mental handicaps’.^5^ Following the inquiries of the late 1960s and early '70s, improvements were made: most of the vast, broken-down Victorian hospitals and asylums eventually closed;^6^ more resources were put into recruiting and training staff and managers;^7^ proper complaints procedures were established;^8^ and, on the back of human rights activism, patient groups were formed and ‘empowered’.^9^

Nevertheless, revelations of cruelty, abuse and neglect have continued to make media headlines with alarming regularity. Furthermore, they have often occurred in places where they are least expected: in hospitals, care homes and clinics charged with a duty of care for some of society’s most vulnerable people – children, older people, people with disabilities. Many of these inquiries, particularly those concerning historical child abuse, return to events that took place in the immediate post-war period. This means that historians can play an important role in augmenting our understanding not only of the socio-political structures that facilitated abusive and neglectful practices in the past, but of the cultural attitudes and belief systems embedded in them.^10^ For example, Shurlee Swain has shown how abusive practices were not only ‘inherent in but essential to’ the way Australian orphanages and children’s homes operated.^11^ Whilst inquiry ‘Chairs’ – many of whom have a legal background – have sought the views of experts from a range of disciplines, historians were rarely included in the past. This has begun to change, particularly in relation to child abuse in Australia, as well as in the Republic of Ireland, the Netherlands and parts of Scandinavia.^12^ In England, Kate Lampard and Ed Marsden sought the expertise of eight historians in order to gain a deeper understanding of the historical culture in which Savile was operating and responses to his behaviour.^13^ Commenting on their final report, historian Sally Sheard observed that ‘it is important that the hospitals in which Savile operated are assessed by the management and patient safety standards of that era, not what we would expect in a 2015 NHS hospital’. She added that to bring about change to systems and processes, staff and patient morale, communication and leadership requires ‘*significant changes to attitudes, values, beliefs and behaviours*’.^14^

Exploring how belief systems become embedded and coded into an institution’s ‘culture’ – its language, systems and practices – became our starting point for the organisation of a cross-disciplinary, international conference titled ‘Cultures of Harm in Institutions of Care’ that was held at Birkbeck, University of London in April 2016. Most of the seven essays in this special issue have emerged from papers that were presented and the discussions that followed.^15^ At the conference and in this issue, we set out to address the discursive shaping and reshaping of cultures that generate and perpetuate, deny and legitimise harmful practices, whether institutionally systemic or perpetrated by an individual – the ‘bad apple’ – or groups of individuals, in sites of care primarily for adults. Essays address practices in England, Ireland, America and Canada during the modern period.

*

First, we should consider definitions. What do we mean by an institution? Geoffrey M. Hodgson offers a broad and workable description, thus: ‘systems of established and embedded social rules that structure social interactions’,^16^ which we explore primarily within the context of bounded communities. The definition evokes sociology theory of the 1950s when, shocked by the appalling conditions in which people were living in psychiatric hospitals, care homes and prisons, social scientists produced a plethora of studies on the social structures that, among other things, facilitated what we would today refer to as ‘abuse’.^17^ Among them was Erving Goffman’s ground-breaking book *Asylums* (1961) in which he described the processes by which institutions stripped inmates of their identity.^18^

Goffman aside, the study of institutions is suffused with the ideas of Michel Foucault who argued that disciplinary systems permeate all social systems thus creating malleable and controllable ‘docile bodies’.^19^ Since then, a vast number of histories have been produced on the social role of institutions within the broader political and welfare systems.^20^ Some have turned their attention towards the internal mechanisms of hospitals and asylums.^21^ This latter focus has been particularly strong among cultural historians, historical archaeologists and historical geographers who explore the mediation of social interactions through the prisms of space, objects and material culture.^22^ Another highly productive avenue of inquiry for historians has been the analysis of oral histories and disclosure narratives of abuse, especially among adults who were abused as children.^23^

Analysts have observed that residential establishments or ‘homes’ are hybrid institutions with poorly defined roles. Ostensibly, they might be called ‘hospitals’, which suggests a medicalised environment; in reality, they provide social care for those unable to look after themselves. They endeavour to create a ‘homely’ atmosphere, but are actually part of a large bureaucratic welfare system in which employees are paid to give ‘compassionate care’ within a structure that is intent on meeting other priorities such as reducing costs.^24^ Privately-run care homes are a good example of the glaring conflict between making a profit and providing high standards of care. Rather than looking at a single type of institution, such as the asylum or the hospital, we examine practices in a range of clinical and quasi-clinical spaces. This, we believe, is a productive way of understanding how social and cultural mechanisms in institutions work. As Jane Hamlett, Lesley Hoskins and Rebecca Preston have argued, cross-institutional studies can make a valuable contribution towards our understanding of broader power structures, the formation of identities and the ongoing reconstitution of environments through the exploration of ‘the *processes* of inhabiting institutions’.^25^

The concept of ‘care’ is more difficult to define because it is used in so many different contexts. As Bernhard Weicht states, the term is ‘deeply personal’. He writes that ‘ideologies, ideas and attitudes about care play an important role in defining the situation and people’s understanding of giving and receiving care’.^26^ Care, particularly formal paid-for care in an institutional setting, involves different levels of interdependency and it is within the interstices of these relationships between the ‘care provider’ and the ‘cared for’ – the social value accorded to each – that unequal and abusive power dynamics can be played out. The word ‘care’ frequently appears in inverted commas denoting its ambivalent nature. For example, in the nineteenth century, doctors were expected to provide ‘reasonable care’, but this was ascertained in lay rather than medical terms.^27^ Historians Catherine Cox and Maria Luddy demonstrate in *Cultures of Care in Irish Medical History* how interests intersect and clash within different traditions as the care people need is compromised and fragmented by a plethora of concerns promulgated by the state, the medical and legal professions, ‘traditional’ healers, the Church, moral reformers and families.^28^

The obvious place to start looking at the role of ‘care’ over time is in the literature on the history of nursing.^29^ In one of the most recent essay collections on the social history of mental health nursing, editors Anne Borsay and Pamela Dale state that ‘the history of care has relatively little to say about paid carers’.^30^ But where is ‘the history of care’? Much has been written about the *provision* – or lack thereof – of social and health care in a range of institutions from hospitals and asylums to workhouses and children’s homes,^31^ many of which address harmful practices.^32^ We agree with Borsay and Dale who argue that we need to move beyond the ‘shackles’ of traditional nursing history, away from Whiggish accounts that draw on stereotypical views of gender, class and race, often embodied in ‘elite figures’.^33^ Daniel J. R. Grey illustrates how those stereotypes are disrupted in his article on nursing ‘character’ in the early twentieth century by drawing attention to a ‘reluctance to acknowledge an unpalatable truth: that a minority of nurses have been – and are – … indifferent to the suffering of patients’.^34^ So, too, does Tommy Dickinson who recounts the cruel process of ‘aversion therapy’ that was used to ‘cure’ homosexuality in psychiatric hospitals in the mid twentieth century. In *‘Curing Queers’* (2015), he exposes a culture of complicity in this painful and humiliating practice by some nurses who were themselves gay, but who simultaneously participated in acts of subversive resistance against doctors.^35^

In this issue, we are interested in the shifting ontological status of ‘care’ and how it is productive of subjective experiences in relation to notions of, for example, ‘vulnerability’, ‘trust’, ‘need’, ‘dependency’ and ‘interdependency’, ‘consent’ and ‘harm’. Indeed, as most of the essays demonstrate, care and harm can co-exist because the same behaviours and attitudes are conceptualised differently depending on the cultural context and perspective of the individual. We cannot ignore the fact that it is so often the least socially valued and most marginalised people who did – and still do – end up in asylums, prisons, reformatories, industrial schools, and long-term residential care homes, having been subjected to different forms of violence often over much of their life course.^36^ As Julia Hallam states, ‘Caring is not just a subjective and material experience but one in which particular historical circumstances, ideologies and power relations create the conditions under which caring can occur, the forms it takes and the consequences it will have for those who undertake it’.^37^ Added to this, we argue, are the consequences for those to whom care is given.

Moving on to the meaning of ‘harm’, we ask if it is the same as violence. Since the 1980s, the meaning of violence in its multiple manifestations has garnered a great deal of attention from historians who contend that over time it may be culturally conceptualised less as the result of a moral failing than as the product of social forces or a particular psychology.^38^ If violence, and in this context harm, involves the transgression of a personal boundary,^39^ it is not difficult to understand how one person’s idea of care is another’s perception of harm given that the former involves the physical and emotional invasion of what Barbara Mortimer terms ‘the private space of others’.^40^ Francisca Loetz argues that ‘what constitutes a “light” or a “serious” injury cannot be established phenomenologically by whether blood is drawn, bones are broken or no physical assault has taken place at all. What counts is the intended and the realized effect on the victims’.^41^

One of the crucial components in the definition of violence is intentionality,^42^ which, as Loetz states, has significant implications in terms of the harm experienced by victims, suggesting that harm is a consequence of violence. The term ‘violence’ is, therefore, too conceptually narrow for our purposes, which is why we have opted to use the word ‘harm’, which does not necessarily imply intentionality**,** since it might come about as the result of error. The existence, or non-existence, of *intention* to harm problematises both the acts themselves and responses to them, particularly when it comes to questions around non-action and neglect, accountability and criminality. Neglect is not passive behaviour. Kim Price writes about ‘active neglect’ in regard to medical negligence in the nineteenth century and, drawing on contemporary work by Tom W. Reader and Alex Gillespie, argues that it ‘easily shrouds the subtle differences (and overlaps) between errors and violations’.^43^ Certain ideologies and systems serve to legitimise violence when, for example, individuals know that they are harming another, but believe that their behaviour is acceptable or even necessary.^44^

Philip Dwyer outlines a useful method for understanding whether violence – or harm – has been done by ‘exploring what, or at what point, a behaviour or a social norm becomes considered transgressive’,^45^ and how that changes over time. In *Men of Blood* (2004), Martin Weiner explains how certain behaviours – such as ‘chastising’ wives – were reconceptualised during the nineteenth century as acts of violence and made more publicly visible by the burgeoning press, thus rendering them less socially acceptable.^46^ However, whether or not harm has been done, intentionally or not, must surely be ascertained from the perspective of the individual against whom it has been perpetrated, rather than from that of the abuser or violator, the agencies that endeavour to pin down whether a crime has been committed, or indeed historians and sociologists. If an individual experiences an act as harmful, then harm has been done.^47^

*

Threaded through all of the topics we address in these essays are ideologies that legitimise harm and mechanisms that perpetuate them through systems of complicity and complacency, normalisation and neutralisation, denial and disavowal at a societal, political, institutional, group and individual level.^48^ One common theme is divided loyalties, which arises time and again among ‘whistle-blowing’ nurses. In this special issue, attention is drawn to doctors working in prisons and for the criminal justice system, where they operate within the fissures of ‘doing no harm’ whilst satisfying the requirements of their paymasters. In ‘Broken Minds and Beaten Bodies’, Catherine Cox and Hilary Marland explain how prison doctors in the 1840s justified the use of the ‘separate system’, despite knowing how harmful it was to prisoners’ mental health, by claiming that prisoners were feigning mental symptoms in order to be transferred to an asylum, where life would be easier. Through processes of labelling and categorising, medical officers and chaplains justified their treatment of vulnerable prisoners. Joanna Bourke focuses on how routine procedures among police surgeons examining female victims of sexual violence required a much higher level of proof and resistance than was necessary by law. At the heart of these practices were long-held beliefs that framed women as devious liars and minimised the harm of sexual violence. She asks: why have attempts to reform medical responses to sexual violence been ineffectual? Louise Hide develops these arguments in her essay on the introduction of mixed-sex wards in psychiatric hospitals from the 1950s showing how, in the new ‘liberalised’ environment of the mid-century, there was more emphasis on the dangers faced by male staff from women maliciously ‘crying rape’ than on female patients who were exposed to ‘sexual psychopaths’ and abusive male staff. Hide contextualises these attitudes within the unintended or ill-considered consequences of the processes that sought to ‘deinstitutionalise’ patients.

Another theme invokes the grainy boundary between punishment and treatment. Leslie Topp’s essay on seclusion in asylum single rooms in the 1830s and ‘40s provides a fascinating counter-point to Cox and Marland’s work on the separate systems in prison. While seclusion in a small room was vaunted as a more humanitarian method of managing disturbed patients than mechanical restraints, it carried uncomfortable parallels with penal practices. Both single rooms and separate cells subjected people to sensory deprivation – or, as Topp suggests, hyper-sensory experiences – leading to debates about the effects of the environment on the mental health of individuals which, ultimately, brought about a change or amelioration of harmful practices. The concept of seclusion, this time from society at large, is also an important theme in ‘A Home or a Gaol’ in which Jennifer Wallis shows how ‘inebriate women’ (who were often assumed to be sexually corrupt) and their ‘polluting’ influence were separated from society in a reformatory, on medical advice. Concepts such as liberty’ and ‘addiction’ were highly gendered and classed in ways that were especially harmful to poor women. Again, we see how harsh treatment, leading to exploitation, was legitimised as ‘reforming’.

Issues around consent and coercion are raised by Whitney Wood and Laura Stark who examine two very different clinical environments in the immediate post-war period. In ‘Put Right Under’, Wood examines how hubristic and hierarchical attitudes in an obstetrics ward led to women being given anaesthetics without their consent. These women were also subjected to painful and humiliating procedures that delayed the birth of their babies until the doctor arrived. Laura Stark shifts the locus of ‘care’ to an American medical research centre in the 1950s where she shows how a legal framework was developed that enabled young Anabaptist men who were conscientious objectors against the Korean War to submit themselves for medical experimentation in the belief that they were undergoing ‘virtuous suffering’. In reality, they were unwittingly participating in a ‘market’ in human subjects. Both of these articles suggest the highly unstable nature of ‘consent’.

*

Whilst writing this introduction, a report into a shocking abuse of power at the Gosport War Memorial Hospital in Hampshire was published. It details events that took place mainly during the 1990s, when the lives of at least 450 older people were ‘shortened’ through administering ‘dangerous’ and unnecessary doses of opioids. Despite repeated calls for an investigation by nursing staff and desperate relatives, the authorities who could have done something – including hospital officials, Hampshire Constabulary, local politicians, the coronial system, the Crown Prosecution Service, the General Medical Council and the Nursing and Midwifery Council – failed to act effectively.^49^ Improvements have been made in hospital care since then, particularly since the publication of the Francis Report in 2013, which focused on safeguarding. But it has still taken 20 years for the families of the people who died at Gosport to have their agonies recognised.

Most depressingly, we must ask what abuses are taking place today that will require future historians to give them their correct cultural context. The separation of thousands of children from their parents at the US-Mexico border will certainly feature as a form of abuse both for the children and their families. The negative impact of ‘austerity’ cuts on public services that have been taking place in the UK, the US, and much of Europe over the past decade will also be analysed by future commentators. Yet, can historians offer more? Many, as Philip Dwyer and Joy Damousi contend, are ‘more comfortable contextualizing violence than theorizing about it’.^50^ How can we push further and deeper, drawing on new theoretic approaches from, for example, the histories of the emotions, the senses and affect, as well as from other academic disciplines, to understand how harm historically has been legitimized, denied and perpetuated in institutions ostensibly dedicated to the ‘care’ of vulnerable peoples?

## Footnotes

1 Kate Lampard and Ed Marsden, *Themes and Lessons Learnt from NHS Investigations into Matters relating to Jimmy Savile. Independent Report for the Secretary of State for Health*, February 2015, 4.

2 The Inquiry was chaired by Robert Francis QC, *Report of the Mid Staffordshire NHS Foundation Trust Public Inquiry*, HC 898 (London: The Stationery Office, 6 February 2013).

3 Department of Health, *Transforming Care: A National Response to Winterbourne View Hospital. Department of Health Review. Final Report.* (December 2012).

4 Among them were The Historical Institutional Abuse Inquiry in Northern Ireland, the Australian Royal Commission into Institutional Child Sexual Abuse, the Independent Jersey Care Inquiry, and the Scottish Inquiry into Historical Child Abuse. A more extensive list can be found in Johanna Sköld and Shurlee Swain, eds, *Apologies and the Legacy of Abuse of Children in* ‘Care’ (Basingstoke: Palgrave Macmillan, 2015).

5 Barbara Robb, *Sans Everything. A Case to Answer. Presented on behalf of AEGIS* (London: Nelson, 1967). See Claire Hilton’s book on her campaign, *Improving Psychiatric Care for Older People. Barbara Robb’s Campaign 1965–1975* (Cham, Switzerland: Palgrave Macmillan, 2017), and J. P. Martin with Debbie Evans, *Hospitals in Trouble* (Oxford: Basil Blackwell, 1984).

6 See Despo Kritsotaki, Vicky Long and Matthew Smith, eds, *Deinstitutionalisation and After. Post-War Psychiatry in the Western World* (Basingstoke: Palgrave Macmillan, 2016); Peter Barham, *Closing the Asylum. The Mental Patient in Modern Society* (London: Penguin, 1992).

7 John Turner *et al*, ‘The History of Mental Health Services in Modern England: Practitioner Memories and the Direction of Future Research’*, Medical History*, 2015, 59, 599-624.

8 See Jonathan Reinarz and Rebecca Wynter, eds, *Complaints, Controversies and Grievances in Medicine. Historical and Social Science Perspectives* (London: Routledge, 2015).

9 See Alex Mold, *Making the Patient-Consumer. Patient Organisations and Health Consumerism in Britain* (Manchester: Manchester University Press, 2015).

10 See Adrian Bingham, Lucy Delap, Louise Jackson & Louise Settle, ‘Historical Child Sexual Abuse in England and Wales: The Role of Historians’, *History of Education*, 45.4, 2016, 411-429.

11 Shurlee Swain, ‘Institutional Abuse: A Long History’, *Journal of Australian Studies*, 42.2, 2018, 153-63.

12 See articles in the special issue of the *Journal of Australian Studies* 42.2, 2018 and the collection of essays co-edited by Swain with Johanna Sköld, *Apologies and the Legacy of Abus*e.

13 Lampard and Marsden, *Themes and Lessons Learnt*, 11, 26. The contribution of historians can be read in the Report; their presentations and supporting texts can be found here: <http://www.historyandpolicy.org/consultations/consultations/jimmy-savile-investigations>, accessed 30 October 2018.

14 Sally Sheard, ‘Can we never learn? Abuse, complaints and inquiries in the NHS’, *History & Policy*, 26 February 2015, our emphasis.

15 Our thanks to the Wellcome Trust and the Birkbeck Wellcome ISSF fund for funding the conference, to Ana Antic for her assistance with organising it, and to all speakers for their invaluable contributions.

16 Geoffrey M. Hodgson, ‘What are Institutions?’, *Journal of Economic Issues*, 40.1, 2006, 1-25, 18.

17 Peter Townsend, *The Last Refuge. A Survey of Residential Institutions and Homes for the Aged in England and Wales* (London: Routledge & Kegan Paul, 1962); Rose Laub Coser, *Life in the Ward* (East Lansing, Michigan State University Press, 1962); Gresham M. Sykes, *The Society of Captives. A Study of a Maximum Security Prison* (Princeton: Princeton University Press, 1958); William Caudill, *The Psychiatric Hospital as a Small Society* (Cambridge, MA: Harvard University Press, 1958); Alfred H. Stanton and Morris S. Schwartz, *The Mental Hospital: A Study of Institutional Participation in Psychiatric Illness and Treatment* (New York: Basic Books, 1954).

18 Erving Goffman, *Asylums*. *Essays on the Social Situations of Mental Patients and Other Inmates* (New York: Anchor Books, 1961).

19 Michel Foucault, *Surveiller et Punir: Naissance de la Prison* (Paris: Éditions Gallimard, 1975); Alan Sheridan (trans.), *Discipline and Punish: The Birth of the Prison* (New York: Pantheon, 1977).

20 Among the most notable is Andrew Scull, *The Most Solitary of Afflictions*. *Madness and Society in Britain 1700-1900* (New Haven and London: Yale University Press, 1993).

21 A small selection includes: Louise Hide, *Gender and Class in English Asylums, 1890-1914* (Basingstoke: Palgrave Macmillan, 2014); David Wright, *Mental Disability in Victorian England. The Earlswood Asylum, 1847-1901* (Oxford: Oxford University Press, 2001); Anne Digby, *Madness, Morality and Medicine. A Study of the York Retreat 1796-1914* (Cambridge: Cambridge University Press, 1985).

22 Leslie Topp, *Freedom and the Cage. Modern Architecture and Psychiatry in Central Europe, 1890-1914* (Pennsylvania: The Pennsylvania State University Press, 2017); Jane Hamlett, *At Home in the Institution. Material Life in Asylums, Lodging Houses and Schools in Victorian and Edwardian England* (Basingstoke: Palgrave Macmillan, 2015); April M. Beisaw and James G. Gibb, eds, *The Archaeology of Institutional Life* (Tuscaloosa: The University of Alabama Press, 2009); Leslie Topp, James E. Moran and Jonathan Andrews, eds, *Madness, Architecture and the Built Environment. Psychiatric Spaces in Historical Contexts* (London: Routledge, 2007); Susan Piddock, *A Space of Their Own. The Archaeology of Nineteenth Century Lunatic Asylums in Britain, South Australia and Tasmania* (New York: Springer, 2007).

23 See Lucy Delap, ‘“Disgusting Details Which are Best Forgotten”: Disclosures of Child Sexual Abuse in Twentieth Century Britain’, *Journal of British Studies*, 57, 2018, 79-107.

24 A more in-depth discussion is in Wendy Parkin and Lorraine Green, ‘Cultures of Abuse within Residential Child Care’, *Early Child Development and Care*, 133.1, 1997, 73-86.

25 Jane Hamlett with Lesley Hoskins and Rebecca Preston, ‘Introduction’, in Jane Hamlett, Lesley Hoskins and Rebecca Preston, eds, *Residential Institutions in Britain, 1725-1970: Inmates and Environments* (London: Pickering & Chatto, 2013), 8, our emphasis.

26 Bernhard Weicht, *The Meaning of Care. The Social Construction of Care for Elderly People* (Basingstoke: Palgrave Macmillan, 2015), 2.

27 Kim Price, *Medical Negligence in Victorian Britain: The Crisis of Care under English Poor Law, c. 1834-1900* (London: Bloomsbury, 2015), 4.

28 Catherine Cox and Maria Luddy, eds, *Cultures of Care in Irish Medical History, 1750-1970* (Basingstoke: Palgrave Macmillan, 2010), Kindle 333.

29 Sue Hawkins, *Nursing and Women’s Labour in the Nineteenth Century: The Quest for Independence* (London: Routledge, 2010); Barbara Mortimer and Susan McGann, eds, *New Directions in the History of Nursing. International Perspectives* (London: Routledge, 2005); Robert Dingwall, Anne Marie Rafferty and Charles Webster, *An Introduction to the Social History of Nursing* (London: Routledge, 1988).

30 Anne Borsay and Pamela Dale, eds, *Mental Health Nursing. The Working Lives of Paid Carers in the Nineteenth and Twentieth Centuries* (Manchester: Manchester University Press, 2015), 1-2.

31 The full historiography is too large to include, but key texts include: Steven King, *Sickness, Medical Welfare and the English Poor, 1750-1834* (Manchester: Manchester University Press, 2018), which includes a chapter on institutions; Jonathan Reinarz and Leonard Schwarz, eds, *Medicine and the Workhouse* (Rochester, NY: University of Rochester Press, 2013); Pat Thane, *Old Age in English History: Past Experiences, Present Issues* (Oxford: Oxford University Press, 2000); Peregrine Horden and Richard Smith (eds), *The Locus of Care. Families, Communities, Institutions, and the Provision of Welfare since Antiquity* (London: Routledge, 1998).

32 See in particular Claudia Malacrida, *A Special Hell: Institutional Life in Alberta’s Eugenic Years* (Toronto: University of Toronto Press, 2015); Vicky Long, ‘Surely a Nice Occupation for a Girl?’ Stories of Nursing, Gender, Violence and Mental Illness in British Asylums, 1914-30’ in Borsay and Dale, eds, *Mental Health Nursing, 123-44*; Geoffrey Reaume, *Remembrance of Patients Past: Patient Life at the Toronto Hospital for the Insane, 1870-1940* (Toronto, Ont; Oxford: Oxford University Press, 2000); Diana Gittins, *Madness in its Place. Narratives of Severalls Hospital, 1913-1997* (London: Routledge 1998).

33 Borsay and Dale, *Mental Health Nursing*, 4.

34 Daniel J. R. Grey, ‘Murder, Mental Illness, and the Question of Nursing “Character” in Early Twentieth Century England’, *History Workshop Journal*, 80, 2015, 183-200, 184-5.

35 Tommy Dickinson, *‘Curing Queers’. Mental Nurses and their Patients, 1935-74* (Manchester: Manchester University Press, 2015).

36 See Price, *Medical Negligence in Victorian Britain*; Alannah Tomkins, *Medical Misadventure in an Age of Professionalisation, 1780-1890* (Manchester: Manchester University Press, 2017).

37 Julia Hallam, ‘Ethical Lives in the Early Nineteenth Century. Nursing and a History of Caring’ in Mortimer and McGann, eds, *New Directions in the History of Nursing*, 22-39, 25.

38 J. Carter Wood, ‘Conceptualizing Cultures of Violence and Cultural Change’, in Stuart Carroll, ed, *Cultures of Violence: Interpersonal Violence in Historical Perspective* (Basingstoke: Palgrave Macmillan, 2007) and Martin J. Weiner, *Reconstructing the Criminal: Culture, Law, and Policy in England, 1830-1914* (Cambridge: Cambridge University Press, 1994).

39 Philip Dwyer, ‘Violence and its Histories: Meanings, Methods, Problems,’ *History and Theory*, Theme Issue 55, (2017), 7-22, 15.

40 Barbara Mortimer, ‘Introduction: The History of Nursing: Yesterday, Today and Tomorrow’ in Mortimer and McGann, eds, *New Directions in the History of Nursing*, 1-21, 13.

41 Francisca Loetz and Rosemary Selle, trans, *A New Approach to the History of Violence. “Sexual Assault” and “Sexual Abuse” in Europe, 1500-1850* (Leiden: Brill, 2015), 9.

42 For a brief discussion of this point, see Dwyer, ‘Violence and its Histories’, 10-11.

43 Price, *Medical Negligence in Victorian Britain, 24.*

44 Dwyer, ‘Violence and its Histories’, 13.

45
*Ibid*, 15.

46 Martin J. Wiener, *Men of Blood: Violence, Manliness, and Criminal Justice in Victorian England* (Cambridge: Cambridge University Press, 2004), 157.

47 For an in-depth discussion of the meaning of sexual violence and the ‘making’ of rapists, see Joanna Bourke, *Rape. A History from 1860 to the Present* (London: Virago, 2007).

48 For an excellent overview of these concepts, see Stanley Cohen, *States of Denial. Knowing about Atrocities and Suffering* (Cambridge: Polity, 2001).

49 The Right Reverend James Jones, KBE, ‘Foreword’, *The Report of the Gosport Independent Panel*, HC 1084 (HMSO: June 2018), viii.

50 Philip Dwyer and Joy Damousi, ‘Theorizing Histories of Violence’, *History and Theory*, Theme Issue 55, 2017, 3-6, 3.

## Notes

Dr Louise Hide is a Wellcome Trust Research Fellow in Medical Humanities. Her research project is titled ‘Cultures of Harm in Residential Institutions for Long-term Adult Care, Britain 1945–1980s’. Her first monograph, *Gender and Class in English Asylums, 1890–1914* was published in 2014. She has published on psychiatric institutions, the ‘patient experience’ and the cultural history of pain and syphilis. Joanna Bourke is Professor of History at Birkbeck, University of London and Fellow of the British Academy. She is the author of 13 books, including *The Story of Pain*. She is the Principal Investigator in a 5-year Wellcome Trust project on ‘Sexual Violence, Medicine and Psychiatry’.

